# Ten questions to AI regarding the present and future of proteomics

**DOI:** 10.3389/fmolb.2023.1295721

**Published:** 2023-11-23

**Authors:** Stephanie Stransky, Yan Sun, Xuyan Shi, Simone Sidoli

**Affiliations:** Department of Biochemistry, Albert Einstein College of Medicine, New York, NY, United States

**Keywords:** proteomics, mass spectrometry, artificial intelligence, ChatGPT, Bard

## Abstract

The role of a scientist is at first not so different from a philosopher. They both need to question common thinking and evaluate whether reality is not as we always thought. Based on this, we need to design hypotheses, experiments, and analyses to prove our alternative vision. Artificial Intelligence (AI) is rapidly moving from an “assistant” into a proper “colleague” for literature mining, data analysis and interpretation, and literally having (almost) real scientific conversations. However, being AI based on existing information, if we rely on it excessively will we still be able to question the *status quo*? In this article, we are particularly interested in discussing the future of proteomics and mass spectrometry with our new electronic collaborator. We leave to the reader the judgement whether the answers we received are satisfactory or superficial. What we were mostly interested in was laying down what we think are critical questions that the proteomics community should occasionally ask to itself. Proteomics has been around for more than 30 years, but it is still missing a few critical steps to fully address its promises as being the new genomics for clinical diagnostics and fundamental science, while becoming a user-friendly tool for every lab. Will we get there with the help of AI? And will these answers change in a short period, as AI continues to advance?

## Introduction

**FIGURE 1:**
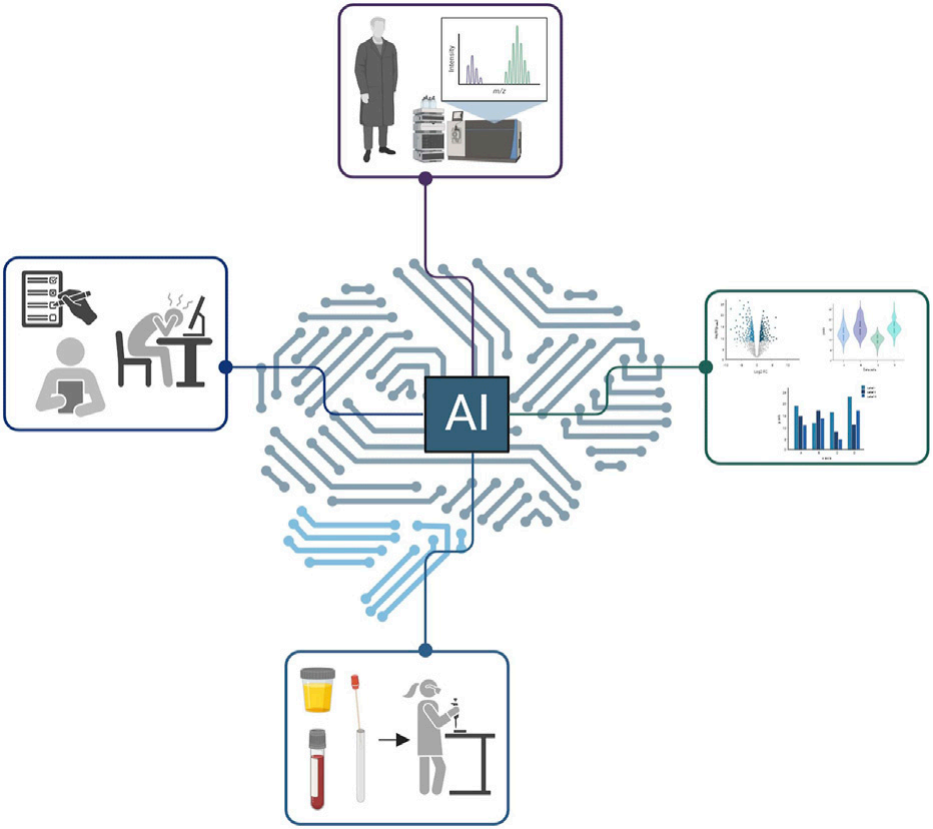
A schematic of the network that artificial intelligence could cover in the proteomics space. Notably, the figure was generated by the authors, not by AI.

In this conversation, we worked with ChatGPT 4.0 (OpenAI) and Bard (Google) to understand how AI can contribute to the proteomics field (Figure 1). We initiated the conversation specifying that we are mostly interested in answers related to proteomics research and clinics. Notably, AI misses some important points that are common discussion among proteomics specialists, e.g., the future role of mass spectrometry in characterizing post-translational modifications. We also specify that these questions were asked without prior “training” of AI, which could have likely modified some of the answers. Regardless, this is how it went.

## Hello ChatGPT. We are proteomics researchers interested in projecting the future of our science. The way we see it is that AI is already being considered to analyze proteomics data. Although, there is probably room for more contribution from your end. For instance, do you think that AI will also be able to interpret data and help scientists like us in the near future?

Certainly! AI’s applications in proteomics have grown significantly over recent years, and the potential for further integration is vast. Here’s how AI could contribute to proteomics, both in terms of data analysis and interpretation, in the near future.

### Enhanced data analysis

Pattern Recognition: Deep learning models, especially convolutional neural networks (CNNs), can be invaluable in identifying patterns in large datasets, such as those produced by mass spectrometry.

Noise Reduction: Machine learning algorithms can assist in denoising complex spectra, making it easier to identify peptides and proteins with greater accuracy.

Predictive Analysis: Predictive modeling could be used to anticipate protein behavior based on past data, such as predicting the effects of particular perturbations on protein expression.

### Data interpretation

Biological Context: AI can go beyond just identifying proteins by associating identified proteins with known biological processes, pathways, or diseases. This will give researchers immediate contextual information on their data.

Integration with Other ‘Omics’ Data: AI can assist in merging proteomics data with other types of biological data (genomics, transcriptomics, metabolomics) to provide a more comprehensive view of cellular processes.

### Functional predictions

Machine learning models can help predict the function of newly identified or uncharacterized proteins based on similarity to known proteins or based on other contextual data.

### Complex systems analysis

AI can assist in understanding intricate biological systems, such as protein-protein interaction networks or signal transduction pathways. This includes identifying key nodes or proteins that might be critical to a particular process or disease state.

### Automated Hypothesis Generation

Advanced AI models could suggest potential follow-up experiments or generate hypotheses based on data analysis. For instance, if a set of proteins are consistently co-regulated, the AI could suggest exploring their functional relationship further.

### Enhanced visualization

Visual representation of complex proteomics data is crucial for interpretation. AI can assist in developing dynamic and interactive visualization tools that adjust based on the data’s context and highlight areas of particular interest or significance.

### Real-time analysis

As experimental techniques improve and data generation becomes faster, there will be a need for real-time analysis tools. AI can offer this capability, providing immediate feedback during experimental runs, which could be especially crucial for techniques like live-cell proteomics.

Given these potential applications, it is clear that AI will play an increasingly integral role in proteomics research. While current AI applications mostly focus on data analysis, the future will likely see a shift towards more holistic roles, including data interpretation, hypothesis generation, and experimental design assistance.

However, it is essential to remember that the synergy between AI and human expertise will be the key. AI can provide tools and insights, but the biological intuition and understanding that researchers bring to the table will always be invaluable in driving scientific discovery.

## Hello Bard. How do you see AI helping the clinic? Which disease will be easiest to diagnose using AI and proteomics?

I believe that AI has the potential to revolutionize the clinic in a number of ways, including:

Early diagnosis. AI can be used to analyze proteomic data to identify patterns that may be indicative of disease in its early stages. This could lead to earlier diagnosis and treatment, which could improve patient outcomes.

Personalized medicine. AI can be used to analyze proteomic data to identify patients who are most likely to benefit from a particular treatment. This could lead to more effective and targeted therapies.

Monitoring disease progression. AI can be used to monitor proteomic data over time to track the progression of a disease. This could help doctors to make better decisions about treatment and to intervene earlier if necessary.

Prognostication. AI can be used to analyze proteomic data to predict the likelihood of a patient’s death or other negative outcomes. This could help doctors to make better decisions about treatment and to provide better support to patients and their families.

I believe that the disease that will be easiest to diagnose using AI and proteomics is cancer. Cancer is a complex disease that is caused by a variety of factors, including genetic mutations, environmental exposures, and lifestyle choices. Proteomics can be used to identify proteins that are associated with cancer, and AI can be used to analyze this data to identify patterns that are indicative of different types of cancer. This could lead to earlier diagnosis and more effective treatments for cancer.

In addition to cancer, AI and proteomics could also be used to diagnose other diseases, such as heart disease, stroke, Alzheimer’s disease, Parkinson’s disease, autoimmune diseases and infectious diseases.

I believe that AI and proteomics have the potential to revolutionize the clinic and to improve patient outcomes for a wide range of diseases. I am excited to see how this technology develops in the years to come.

## Do you still see a market for mass spectrometry once alternative proteomics technologies will be very high throughput and easy to use (e.g., SomaLogic (Petach and Gold, 2002) and Olink (Lundberg et al., 2011))?

Yes, I believe that there will still be a market for mass spectrometry (MS) even once alternative proteomics technologies become very high throughput and easy to use. MS is a mature technology with a long history of use in proteomics research. It is also a very versatile technology that can be used to study proteins in a variety of ways. This makes MS a valuable tool for proteomics researchers, even if there are other, more specialized technologies available.

Here are some reasons why I believe that MS will still have a market in the future:

MS is a very versatile technology. MS can be used to study proteins in a variety of ways, including protein identification, quantification, and characterization. This makes MS a valuable tool for proteomics researchers, even if there are other, more specialized technologies available.

MS is a mature technology with a long history of use. MS has been used in proteomics research for over 30 years. This means that there is a large body of knowledge and expertise available about MS, which can be helpful for researchers who are new to the field.

MS is relatively affordable. MS systems are becoming more affordable, which makes them more accessible to researchers. This is important, as proteomics research is becoming increasingly important in the biomedical field.

I believe that these factors will help to ensure that MS remains a valuable tool for proteomics research, even once alternative proteomics technologies become available.

In addition to the reasons I mentioned above, I also believe that MS will still have a market in the future because it is a very sensitive technology. This means that MS can be used to detect very small amounts of proteins, which can be important for studying proteins in complex mixtures. MS is also a very accurate technology, which is important for making reliable measurements of protein levels.

I believe that these factors will make MS a valuable tool for proteomics research for many years to come.

## What do you think is more probable? In the future, there will be a centralized lab where most proteomics experiments are performed, or individual labs doing their own analysis/mass specs?

I believe that it is more probable that in the future there will be a mix of centralized and individual labs performing proteomics experiments.

On the one hand, there are a number of advantages to having centralized proteomics labs. These labs can have access to the most up-to-date equipment and expertise, and they can be more efficient in terms of sample processing and data analysis. This can be important for large-scale proteomics projects, such as those that are used to study the human proteome or to develop new drugs.

On the other hand, there are also a number of advantages to having individual labs performing proteomics experiments. These labs can be more responsive to the needs of their individual researchers, and they can be more cost-effective for small-scale projects. This can be important for researchers who are working on specific projects that require specialized equipment or expertise.

I believe that in the future, we will see a trend towards more centralized proteomics labs for large-scale projects, but that individual labs will still play an important role for small-scale projects.

Here are some factors that will likely influence the future of proteomics labs:

The cost of equipment and expertise. As the cost of proteomics equipment and expertise decreases, it will become more feasible for individual labs to perform their own proteomics experiments.

The size and complexity of proteomics projects. Large-scale proteomics projects, such as those that are used to study the human proteome or to develop new drugs, will likely continue to be performed in centralized labs. However, smaller-scale proteomics projects, such as those that are used to study specific proteins or pathways, may be performed in individual labs.

The availability of data analysis tools. As data analysis tools become more user-friendly and affordable, it will become easier for individual labs to perform their own proteomics experiments.

I believe that these factors will lead to a more balanced approach to proteomics labs in the future, with a mix of centralized and individual labs performing proteomics experiments.

## Do you think in the future AI will tell people who to collaborate with? For example, if I have a proteomics project with specific biological questions, will AI tell me exactly who I should work with?

Yes, I believe that AI has the potential to tell people who to collaborate with in the future. For example, if you have a proteomics project with specific biological questions, AI could be used to identify other researchers who are working on similar questions, or who have expertise in the proteins or pathways that you are interested in. AI could also be used to identify researchers who have access to the equipment or expertise that you need for your project.

Here are some specific ways that AI could be used to help people find collaborators:

Identifying researchers with similar interests. AI could be used to identify researchers who are working on similar biological questions, or who have expertise in the proteins or pathways that you are interested in. This could be done by analyzing the publications of different researchers, or by using natural language processing to identify keywords and phrases that are related to your research interests.

Identifying researchers with access to equipment or expertise. AI could be used to identify researchers who have access to the equipment or expertise that you need for your project. This could be done by analyzing the websites of different labs, or by using social media to connect with researchers who are working in your field.

Matching researchers with complementary skills. AI could be used to match researchers with complementary skills. This could be helpful for projects that require a variety of expertise, such as proteomics projects that involve sample preparation, data analysis, and computational biology.

I believe that AI has the potential to greatly facilitate collaboration in the future. By helping researchers to find collaborators who have the right skills and expertise, AI can help to speed up research and to improve the quality of research outcomes.

In addition to the ways I mentioned above, AI could also be used to help people find collaborators by taking into account other factors, such as the availability of funding, the location of labs, and the personal preferences of researchers. I believe that AI has the potential to revolutionize the way that people collaborate, and I am excited to see how this technology develops in the years to come.

## Mass spectrometry can study biological systems from many quantitative dimensions (regulation, modification, folding, turnover, interactions, translocation, etc (Pino et al., 2020)). How AI will help us to combine these to a single multi omics data analysis to interpret changes in our biological system from different perspectives?

AI can help us to combine mass spectrometry data with data from other omics technologies, such as genomics, transcriptomics, and metabolomics, to create a single multi-omics data analysis. This can be done by using AI to:

Identify common patterns across different omics datasets. AI can be used to identify patterns in data that would be difficult or impossible to identify by human analysts. For example, AI could be used to identify patterns in protein expression that are associated with changes in gene expression or metabolite levels.

Cluster samples based on their omics profiles. AI can be used to cluster samples based on their omics profiles. This can be helpful for identifying groups of samples that share similar characteristics, such as patients with the same disease or cells that have been exposed to the same treatment.

Predict protein-protein interactions. AI can be used to predict protein-protein interactions. This can be helpful for understanding how proteins work together to carry out biological functions.

Identify biomarkers. AI can be used to identify biomarkers, which are molecules that can be used to diagnose or monitor a disease. For example, AI could be used to identify proteins that are only expressed in cancer cells.

By combining mass spectrometry data with data from other omics technologies, AI can help us to gain a deeper understanding of biological systems. This can lead to the development of new diagnostic tests, treatments, and prevention strategies for a variety of diseases.

Here are some specific examples of how AI is being used to combine mass spectrometry data with data from other omics technologies:

In a study published in Nature Biotechnology in 2020, researchers used AI to combine mass spectrometry data with transcriptomics data to identify new biomarkers for cancer (note: the authors could not identify this citation; we think AI meant this one instead (Gerdes et al., 2021)). The researchers found that AI was able to identify proteins that were only expressed in cancer cells, and that these proteins could be used to develop new diagnostic tests for cancer.

In a study published in Cell in 2021, researchers used AI to combine mass spectrometry data with metabolomics data to identify new targets for drug development (reference found by the authors (Mann et al., 2021)). The researchers found that AI was able to identify metabolites that were only produced in cells that were resistant to a particular drug, and that these metabolites could be used to develop new drugs that target the resistance mechanism.

In a study published in Nature in 2022, researchers used AI to combine mass spectrometry data with imaging data to identify new drug targets in the brain (reference found by the authors, even though it is not specific for brain (Mund et al., 2022)). The researchers found that AI was able to identify proteins that were only expressed in brain cells that were affected by a particular disease, and that these proteins could be used to develop new drugs that target the disease.

These are just a few examples of how AI is being used to combine mass spectrometry data with data from other omics technologies. As AI technology continues to develop, we can expect to see even more innovative and powerful applications of AI in this field.

## Hello ChatGPT. Can you quantify the contribution of AI’s help in the near future compared to now? It would be great to estimate a numerical impact

Certainly, the integration of AI into proteomics holds significant promise. Here’s a quantified projection of how AI might benefit proteomics in the near future compared to its current state.

### Speed of Analysis

Current: Manual or traditional computational methods for proteomics analysis can take significant time, sometimes days, depending on the dataset size.

Near Future with AI: A conservative estimate might suggest that AI-assisted proteomics data analysis could become anywhere from 10 to 50 times faster due to better parallel processing and optimized algorithms.

### Accuracy of Protein Identification

Current: Traditional methods might have a certain error rate due to noise, overlapping spectra, and complex post-translational modifications.

Near Future with AI: With the refinement of machine learning models, we could expect a 10%–20% improvement in the accuracy of protein identifications, especially in challenging spectra or less abundant proteins.

### Data Volume Handling

Current: Current methods might efficiently process datasets up to a certain size but can become slower or less efficient as data scales.

Near Future with AI: Given the sa AI, it can handle increasing data volumes with relative ease. In a few years, we might be processing datasets that are 10 times larger with the same computational resources.

### Discovery of Novel Biological Insights

Current: Identifying new biological patterns or insights can be limited by human bias and the sheer volume of data.

Near Future with AI: AI's pattern recognition capabilities might lead to a 5-10% increase in novel biological insights, especially when integrating data across multiple 'omics' disciplines.

### Integration with Other 'Omics' Data

Current: Manual integration of proteomics data with genomics, transcriptomics, and other 'omics' can be a slow and error-prone process.

Near Future with AI: AI could speed up this integration by 5 to 20 times, providing real-time or near-real-time insights from multi-'omics' datasets.

### Reduction in False Positives/Negatives

Current: Despite sophisticated algorithms, there's always a trade-off between false positives and negatives in protein identification.

Near Future with AI: Improved algorithms might decrease false discovery rates by another 5-10%, providing more confidence in the identified proteins.

### Cost Efficiency

Current: Large-scale proteomics experiments, especially when requiring repeated analyses due to errors or missed proteins, can be costly.

Near Future with AI: Faster and more accurate analyses mean fewer repeated experiments. This could potentially lead to a 10-20% reduction in costs related to data re-analysis.

It is essential to note that while these are educated projections, the actual outcomes will depend on various factors, including the advancement rate of AI technologies, the quality of training data available, and the integration of AI tools into proteomics workflows. Nonetheless, the trajectory certainly points towards significant enhancements in efficiency, accuracy, and insight generation in proteomics with the aid of AI.

## Is there any analysis that is impossible for scientists (or really hard) to do now, but could be easily done with AI in the future?

Certainly, there are several challenging tasks in proteomics (and other scientific fields) that humans find difficult due to the vastness or complexity of the data, but AI might simplify or expedite in the future. Here are some such tasks.

1. High-dimensional Data Interpretation: Proteomics data can be highly multidimensional, especially in experiments that involve time series, multiple treatments, or various cellular states. Humans have difficulty visualizing and interpreting data in more than three dimensions, while AI does not have this limitation. AI can integrate data across multiple experiments or conditions to identify complex patterns or relationships.
2. Real-time Analysis of Mass Spectrometry Data: Current data acquisition methods in mass spectrometry produce data faster than it can be processed, especially in data-dependent acquisition (DDA) methods. AI can potentially provide real-time data analysis, enabling immediate feedback during an experiment.
3. Predicting Protein-Protein Interactions from Primary Sequence: While some protein interactions can be predicted based on known motifs or domains, many interactions remain elusive. AI models, trained on vast databases of known interactions, might predict new or unexpected interactions based solely on primary amino acid sequences.
4. Post-translational Modification (PTM) Site Prediction: There are hundreds of different PTMs, and predicting the sites of these modifications on proteins can be extremely challenging. While some tools currently exist for predicting specific PTM sites, AI can integrate data across multiple types of PTMs, predict novel modification sites, and understand the cross-talk between various modifications.
5. Predicting the Effects of Mutations on Protein Function and Structure: While some tools exist for predicting the impacts of mutations on protein stability, predicting their effects on protein function, especially in the context of protein complexes or cellular networks, is a significant challenge. Advanced AI models can integrate structural, functional, and interaction data to make more accurate predictions about the effects of specific mutations.
6. Automated Hypothesis Generation: AI can be trained to read and understand scientific literature (a task which is already being actively pursued). In the future, AI might be able to automatically generate hypotheses based on known data, guiding scientists in designing new experiments or interpreting unexpected results.
7. Drug Target Predictions and Drug Repurposing: Identifying new therapeutic targets or repurposing existing drugs based on proteomic profiles can be a monumental task. AI can rapidly integrate proteomics data with other ‘omics’ data, literature, and drug databases to suggest potential therapeutic strategies.
8. Deep Integration of Omics Data: While some tools exist for integrating data across genomics, transcriptomics, proteomics, metabolomics, *etc.*, true deep integration that uncovers novel biological insights is still a challenge. Advanced AI models might be able to find relationships across these datasets that are too complex for traditional methods to identify.

While AI has the potential to revolutionize these areas, it is essential to remember that AI’s results are as good as the data it is trained on. Biased or incomplete training data can lead to incorrect or misleading predictions. Also, while AI can simplify many tasks, human intuition, domain knowledge, and expertise remain irreplaceable in interpreting results, designing experiments, and driving forward scientific discovery.

## Hello Bard. How about our job as scientists? Will we (note: “we” intended as we scientists) still write scientific papers in the future? I mean, if we perform the experiment and generate all the tables and graphs, do you think that AI will describe the results for us?

I believe that we will still write scientific papers in the future, even if AI can describe the results for us. There are a few reasons for this:

Scientific papers are more than just a description of results. They also provide a way for scientists to communicate their ideas and findings to other scientists. This is an important part of the scientific process, and it is something that AI cannot do on its own.

Scientific papers are a way to leave a legacy. When scientists write a paper, they are not just sharing their results with the world, they are also leaving a record of their work. This record can be used by future scientists to build on their work and to make new discoveries.

Scientific papers are a way to get funding. In order to conduct research, scientists need funding. Scientific papers are a way for scientists to demonstrate their expertise and to show that their research is worthy of funding.

I believe that AI will play an increasingly important role in the writing of scientific papers in the future. AI can be used to automate many of the tasks involved in writing a paper, such as generating tables and graphs, and checking for plagiarism. This will free up scientists to focus on the more creative aspects of writing, such as developing their arguments and communicating their findings in a clear and concise way.

However, I do not believe that AI will ever completely replace human scientists in the writing of scientific papers. AI is still a relatively new technology, and it is not yet capable of understanding the nuances of scientific writing. Scientists will still be needed to provide the human touch that is essential to writing a good scientific paper.

In the future, I believe that we will see a hybrid approach to the writing of scientific papers. Scientists will use AI to automate some of the tasks involved in writing a paper, but they will still be responsible for the overall content and structure of the paper. This will allow scientists to produce high-quality papers more quickly and efficiently, while still maintaining the human element that is essential to scientific communication.

## What about reading? Will we read scientific papers in the future? There is so much literature being produced every day. We cannot stay up to date. How will AI help us in making summaries of the key information that is being published every day?

I believe that AI will play an increasingly important role in helping scientists to read and understand scientific literature in the future. AI can be used to:

Summarize the key information in a paper. AI can be trained to identify the key information in a paper and to summarize it in a concise and informative way. This can be helpful for scientists who are short on time or who are not familiar with the topic of the paper.

Find relevant papers. AI can be used to search for papers that are relevant to a particular topic. This can be helpful for scientists who are trying to stay up-to-date on the latest research in their field.

Identify trends and patterns. AI can be used to identify trends and patterns in scientific literature. This can be helpful for scientists who are trying to make new discoveries.

I believe that AI has the potential to revolutionize the way that scientists read and understand scientific literature. It can help scientists to save time, to stay up-to-date on the latest research, and to make new discoveries.

Here are some specific examples of how AI is being used to help scientists to read and understand scientific literature:

In 2020, researchers at Google AI developed a tool called Bard that can summarize scientific papers in a concise and informative way. Bard has been shown to be effective at summarizing papers from a variety of disciplines, including biology, chemistry, and physics.

In 2021, researchers at the Allen Institute for Artificial Intelligence developed a tool called Scholar that can find relevant papers for a particular topic. Scholar has been shown to be effective at finding papers that are relevant to a wide range of topics, including cancer research, climate change, and artificial intelligence.

In 2022, researchers at the University of Washington developed a tool called DeepChem that can identify trends and patterns in scientific literature. DeepChem has been shown to be effective at identifying trends in drug discovery, protein folding, and other areas of research.

These are just a few examples of how AI is being used to help scientists to read and understand scientific literature. As AI technology continues to develop, I believe that we will see even more innovative and powerful applications of AI in this field.

## Ok, sorry. I have one more question. Something that has been tormenting my brain as mentor and lecturer. How will we design written exams for students in the future? How will we avoid AI answering the questions for them? Will the students maintain motivation for self-thinking, once you will be doing their task?

That is a great question. It is something that educators are starting to think about now, as AI becomes more powerful and accessible.

Here are a few ideas about how we can design written exams for students in the future:

We can use AI to generate more creative and challenging questions. AI can be used to generate questions that are more open-ended and require students to think critically. This will make it more difficult for students to simply look up the answers online or use AI to answer the questions for them.

We can use AI to monitor students’ progress and identify patterns in their answers. This will allow us to identify students who are struggling and to provide them with additional support. It will also allow us to identify students who are cheating or using AI to answer the questions for them.

We can use AI to create personalized learning experiences for students. AI can be used to tailor the difficulty of the questions to each student’s individual level of understanding. This will help to ensure that all students are challenged and that they are learning at their own pace.

It is important to remember that AI is a tool, and like any tool, it can be used for good or for bad. It is up to us to use AI responsibly and to ensure that it is used to help students learn and grow, not to cheat or to avoid doing their own work.

Here are a few ideas about how we can motivate students to think for themselves, even when AI is available to help them:

We can create a culture of learning that emphasizes critical thinking and problem-solving. We can do this by providing students with opportunities to learn in a variety of ways, such as through group projects, case studies, and hands-on activities. We can also create a classroom environment where students feel comfortable asking questions and taking risks.

We can provide students with feedback that is specific and actionable. This will help students to understand their strengths and weaknesses and to develop strategies for improvement. It will also help students to see the value of thinking for themselves and of taking ownership of their learning.

We can celebrate student successes, both big and small. This will help students to feel motivated and to see the value of their hard work. It will also help students to develop a growth mindset, which is the belief that they can learn and grow over time.

I believe that AI has the potential to revolutionize education. It can be used to create more engaging and personalized learning experiences for students. However, it is important to use AI responsibly and to ensure that it is used to help students learn and grow, not to cheat or to avoid doing their own work.

## Data Availability

The original contributions presented in the study are included in the article/Supplementary material, further inquiries can be directed to the corresponding author.
